# Frequency of Positive Conversion of Interferon-Gamma Release Assay Results Among Patients With Inflammatory Bowel Disease Treated With Non-tumor Necrosis Factor Inhibitors

**DOI:** 10.3389/fmed.2021.670242

**Published:** 2021-05-21

**Authors:** Kyuwon Kim, Kyung-Wook Jo, Tae Sun Shim, Jin Hwa Park, Sung Wook Hwang, Sang Hyoung Park, Dong-Hoon Yang, Jeong-Sik Byeon, Seung-Jae Myung, Suk-Kyun Yang, Byong Duk Ye

**Affiliations:** ^1^Department of Gastroenterology, Asan Medical Center, University of Ulsan College of Medicine, Seoul, South Korea; ^2^Department of Pulmonology and Critical Care Medicine, Asan Medical Center, University of Ulsan College of Medicine, Seoul, South Korea; ^3^Inflammatory Bowel Disease Center, Asan Medical Center, University of Ulsan College of Medicine, Seoul, South Korea

**Keywords:** inflammatory bowel disease, latent tuberculosis infection, interferon-gamma release assay, vedolizumab, ustekinumab

## Abstract

Considering the risk of reactivation of latent tuberculosis infection (LTBI), not only before starting tumor necrosis factor inhibitors but also before non-TNF inhibitor therapy, LTBI screening is routinely recommended for patients with inflammatory bowel disease (IBD). However, data on the positive conversion of LTBI test results during non-TNF inhibitor therapy are scarce. Among IBD patients treated with vedolizumab and/or ustekinumab, a total of 91 patients who had negative baseline interferon-gamma release assay (IGRA) results, assessed by QuantiFERON^®^-TB Gold In-tube or QuantiFERON^®^-TB Gold Plus, were enrolled. Serial LTBI test results after starting non-TNF inhibitor therapy were collected, and patients' clinical characteristics were analyzed. Positive IGRA conversion was observed in six of 91 patients (6.6%). The cumulative IGRA conversion–free survival rates after starting therapy were 97.7% after 1 year and 86.7% after 2 years. Ulcerative colitis was more common among converters compared with non-converters (66.7 vs. 23.5%, *P* = 0.040). Among six converters, four had been treated with vedolizumab, one with ustekinumab, and the other with vedolizumab followed by ustekinumab. All six patients had been previously exposed to TNF inhibitors before non-TNF inhibitor therapy: five to infliximab and one to both infliximab and adalimumab. After positive IGRA conversion, none of the six converters developed active tuberculosis while maintaining non-TNF inhibitor therapy (median 6.8 months, range 0.4–32.1 months). Positive IGRA conversion among IBD patients treated with vedolizumab and/or ustekinumab appears to occur somewhat frequently, but its clinical implications remain to be elucidated.

## Introduction

The emergence of TNF (tumor necrosis factor) inhibitors has brought about significant improvements in clinical outcomes for patients with inflammatory bowel disease (IBD) refractory to conventional treatment. Despite the remarkable effectiveness of TNF inhibitors, however, reactivation of latent tuberculosis (TB) is a major safety concern. The risk of TB development among IBD patients treated with TNF inhibitors has been reported to increase 1.6- to 41.7-fold, depending on regional variations in TB burden and baseline risk (1, 2). Accordingly, the consensus guidelines of the European Crohn's and Colitis Organization (ECCO) (3) and the Asian Organization for Crohn's and Colitis (AOCC) and the Asia Pacific Association of Gastroenterology (APAGE) (4) recommend screening for TB and treating latent TB infection (LTBI) before the initiation of TNF inhibitor therapy.

Meanwhile, non-TNF inhibitors such as vedolizumab and ustekinumab, have shown their efficacies for inducing and maintaining clinical response and remission in patients with moderate-to-severe IBD (5–8). Owing to their mechanisms of action, vedolizumab and ustekinumab could be regarded as safer than TNF inhibitors in terms of LTBI reactivation and active TB development. In the IM-UNITI study, only one (0.25%) out of 397 patients developed active TB (7); likewise, a recent descriptive analysis of the 4 years post-marketing safety data based on the Vedolizumab Global Safety Database (VGSD) reported 9 (0.03%) events of TB in 208,050 patient-years of vedolizumab exposure (9). Hence, it is anticipated that the risk of TB is lower among IBD patients treated with vedolizumab or ustekinumab compared with those treated with TNF inhibitors. As the development of TB among patients treated with TNF inhibitors is predominantly through LTBI reactivation (10), Lee et al. (11) investigated the LTBI test conversion rate and the subsequent risk of TB during TNF inhibitor therapy. However, data on positive LTBI test result conversion among patients treated with non-TNF inhibitors are scarce. Therefore, we now report the frequency of positive conversion of interferon-gamma release assay (IGRA) results among IBD patients treated with non-TNF inhibitors, such as vedolizumab and ustekinumab, in an area with an intermediate TB burden.

## Methods

We performed a retrospective observational study including patients with IBD who had been treated with vedolizumab and/or ustekinumab at the Asan Medical Center, a tertiary-care teaching hospital in South Korea, between August 2017 and December 2020. During this period, patients who had negative baseline IGRA results by QuantiFERON^®^-TB Gold In-tube (QFT-GIT; QIAGEN, Hilden, Germany), or QuantiFERON^®^-Gold Plus (QFT-Plus; QIAGEN, Hilden, Germany) were included. Vedolizumab was prescribed for patients with active Crohn's disease (CD) or ulcerative colitis (UC) and ustekinumab for patients with active CD, according to the approved indication of drugs in Korea. In total, 91 eligible patients were enrolled in our study. Among them, 11 patients had a history of LTBI and eight had a history of pulmonary TB prior to non-TNF inhibitor therapy. All patients were included in the study regardless of the history related to TB considering that (1) there is a possible risk of reinfection of TB for the residents in Korea, a country with an intermediate TB burden, and (2) the adequacy of TB treatment was not thoroughly documented in the medical records of some patients. Following the prospective registry protocol, IGRAs were performed at 30, 54, and 110 weeks after vedolizumab initiation, and at 26, 52, and 104 weeks after ustekinumab initiation. However, some IGRAs deviated from the above protocol, and those performed at the physician's discretion were also collected and analyzed. Categorical variables were expressed as numbers and percentages, and continuous variables were expressed as median and interquartile range (IQR). The crude and cumulative rates of positive IGRA conversion were calculated, and the patients' clinical characteristics were analyzed and compared according to IGRA conversion. For comparisons between IGRA positive converters and non-converters, the chi-squared test was used for categorical variables and the Mann-Whitney *U*-test was used for continuous variables. The ethics committee of Asan Medical Center approved the study protocol (IRB Number: 2021-0058).

## Results

**Figure 1:**
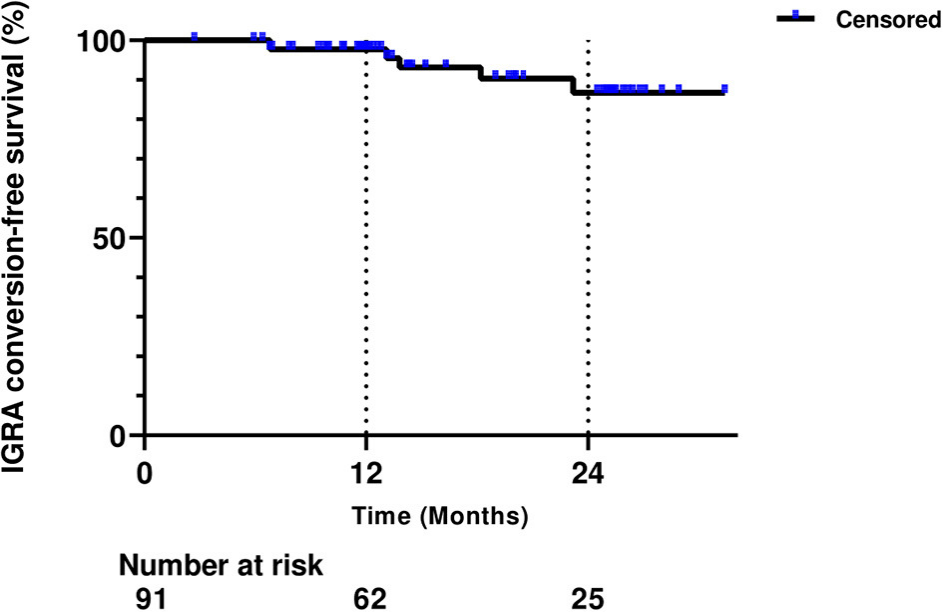
Cumulative interferon-gamma release assay (IGRA) conversion-free survival rate after starting non-tumor necrosis factor inhibitor therapy.

Out of 91 patients, 55 were males (60.4%), and the median age at baseline was 36 years (interquartile range [IQR], 29–45.8 years) (Table 1). Sixty-seven patients (73.6%) had CD, and the median disease duration was 11.3 years (IQR, 8.1–17.0 years). A total of 89 patients (97.8%) had a previous history of exposure to TNF inhibitors. Vedolizumab, ustekinumab, and ustekinumab after vedolizumab failure were given to 47 (51.6%), 23 (25.3%), and 21 (23.1%) patients, respectively. The median duration of follow-up after non-TNF inhibitor commencement was 22.3 months (IQR, 15.5–31.1 months). During follow-up, six patients (6.6%) underwent positive IGRA conversion (Table 1). The proportion of patients with UC was significantly different between converters and non-converters (66.7 vs. 23.5%, *P* = 0.040), whereas other characteristics were not different between the two groups (Table 1). The cumulative IGRA conversion-free survival rate after starting non-TNF inhibitor therapy was 97.7% after 1 year and 86.7% after 2 years (Figure 1).

**Table 1:** Demographic and clinical characteristics of patients.

	**Converters (*n* = 6)**	**Non-converters (*n* = 85)**	**Total (*n* = 91)**	***P***
Age, median (IQR), years	40 (33.8–54.5)	35 (29.0–44.0)	36 (29.0–45.8)	0.222
**Sex**, ***n*** **(%)**				
Male	4 (66.7)	51 (60.0)	55 (60.4)	0.747
Female	2 (33.3)	34 (40.0)	36 (39.6)	
**IBD type**, ***n*** **(%)**				
UC	4 (66.7)	20 (23.5)	24 (26.4)	0.040
CD	2 (33.3)	65 (76.5)	67 (73.6)	
Disease duration, median (IQR), years	5.0 (4.4–17.3)	11.5 (8.6–17.1)	11.3 (8.1–17.0)	0.168
Follow-up duration after non-TNF inhibitor therapy, median (IQR), months	22.3 (17.3–37.3)	22.3 (15.3–30.6)	22.3 (15.5–31.1)	0.473
**Previous TNF inhibitors**, ***n*** **(%)**				
None	0 (0.0)	2 (2.4)	2 (2.2)	0.987
IFX	5 (83.3)	54 (63.5)	59 (64.8)	
ADM	0 (0.0)	8 (9.4)	8 (8.8)	
GLM	0 (0.0)	1 (1.2)	1 (1.1)	
IFX, ADM	1 (16.7)	17 (20.0)	18 (19.8)	
ADM, GLM	0 (0.0)	1 (1.2)	1 (1.1)	
IFX, GLM	0 (0.0)	1 (1.2)	1 (1.1)	
IFX, ADM, GLM	0 (0.0)	1 (1.2)	1 (1.1)	
**Concomitant IST**, ***n*** **(%)**				
Yes	4 (66.7)	53 (62.4.)	57 (62.6)	>0.999
No	2 (33.3)	32 (37.6)	34 (37.4)	
**Concomitant corticosteroids**, ***n*** **(%)**				
Yes	2 (33.3)	11 (12.9)	13 (14.3)	0.203
No	4 (66.7)	74 (87.1)	78 (85.7)	
**Non-TNF inhibitor**, ***n*** **(%)**				
VED	4 (66.7)	43 (50.6)	47 (51.6)	0.747
UST	1 (16.7)	22 (25.9)	23 (25.3)	
UST after VED	1 (16.7)	20 (23.5)	21 (23.1)	
**Previous LTBI or TB treatment**, ***n*** **(%)**				
Yes	2 (33.3)^a^	9 (10.6)	11 (12.1)	0.152
No	4 (66.7)	76 (89.4)	80 (87.9)	

*IQR, interquartile range; IBD, inflammatory bowel disease; UC, ulcerative colitis; CD, Crohn's disease; TNF, tumor necrosis factor; IFX, infliximab; ADM, adalimumab; GLM, golimumab; IST, immunosuppressive therapy; VED, vedolizumab; UST, ustekinumab; LTBI, latent tuberculosis infection; TB, tuberculosis*.

a *Excluding one patient who was given a standard anti-tuberculosis therapeutic trial for 11 weeks for differentiation between Crohn's disease and intestinal tuberculosis*.

The characteristics of the six IGRA converters are summarized in Table 2. Among the six converters, four had been treated with vedolizumab, one with ustekinumab, and the other with vedolizumab followed by ustekinumab. The median interval from non-TNF inhibitor commencement to IGRA conversion was 13.5 months (range, 6.8–23.2 months). Five converters had been previously exposed to infliximab and one to both infliximab and adalimumab. Two patients (Patient 3 and Patient 6) received LTBI treatment after confirmation of IGRA conversion, but none discontinued non-TNF inhibitors. After positive IGRA conversion, none of the six converters developed active TB while maintaining non-TNF inhibitor therapy (median 6.8 months, range 0.4–32.1 months).

**Table 2:** Demographic and clinical characteristics of IGRA converters among IBD patients treated with non-TNF inhibitors.

	**Patient 1**	**Patient 2**	**Patient 3**	**Patient 4**	**Patient 5**	**Patient 6**
Age/sex	33/M	50/F	36/F	68/M	44/M	34/M
IBD type	CD	UC	UC	UC	UC	CD
Disease duration, years	16.7	4.4	4.3	5.0	4.9	19.0
Baseline chest radiograph	Normal	Normal	Normal	Normal	Normal	Normal
Baseline QFT-GIT or QFT-Plus^a^ result (IU/ml)	−0.38	0.27	0.07	0.04	−0.06, −0.03^a^	0.03, 0.05^a^
Converted QFT-GIT or QFT-Plus^a^ result (IU/ml)	0.60	0.92	0.55	0.17, 0.39^a^	2.20, 1.61^a^	0.38, 0.55^a^
Non-TNF inhibitors administered	UST after VED	VED	VED	VED	VED	UST
Interval from non-TNF inhibitor commencement to positive IGRA conversion, months	6.8	13.1	6.8	23.2	13.8	18.2
Previous history of TB	No	No	No	TB pleurisy	No	No
Previous TB treatment	Yes^b^	No	No	Yes^c^	No	No
Previous LTBI treatment	No	No	No	No	Yes	No
Previous TNF inhibitors before non-TNF inhibitor therapy	IFX, ADM	IFX	IFX	IFX	IFX	IFX
Concomitant IST	MTX	No	AZAT	No	AZAT	AZAT
Concomitant corticosteroids	No	Yes	No	Yes	No	No
LTBI treatment after IGRA conversion	No	No	Yes	No	No	Yes
Interruption of non-TNF inhibitors due to IGRA conversion	No	No	No	No	No	No
Maintenance duration of non-TNF inhibitors after IGRA conversion, months	32.1	23.6	11.0	0.4	1.8	2.6
Development of active TB	No	No	No	No	No	No

*IGRA, interferon-γ release assay; IBD, inflammatory bowel disease; TNF, tumor necrosis factor; M, male; F, female; CD, Crohn's disease; UC, ulcerative colitis; UST, ustekinumab; VED, vedolizumab; TB, tuberculosis; LTBI, latent tuberculosis infection; IFX, infliximab; ADM, adalimumab; IST, immunosuppressive therapy; MTX, methotrexate; AZAT, azathioprine*.

a *QuantiFERON^®^-TB Gold Plus result consists of antigen responses from both TB_1_ and TB_2_. The first value represents response from TB_1_ and the second from TB_2_*.

b *Standard anti-tuberculosis therapeutic trial for 11 weeks for differentiation between Crohn's disease and intestinal tuberculosis*.

c *Standard anti-tuberculosis treatment for 9 months for tuberculous pleurisy*.

## Discussion

In this study, six patients (6.6%) developed positive IGRA conversion in a median of 13.5 months after initiating non-TNF inhibitors; however, none of the converters developed active TB during a median follow-up of 6.8 months despite continued non-TNF inhibitor treatment. According to previous studies on LTBI and TB monitoring during TNF inhibitor therapy, fifteen out of 78 patients (19.2%) with IBD experienced positive conversion of any LTBI tests after a median follow-up of 16 months in Hong Kong (11), and 11.8% of patients with rheumatologic diseases underwent positive IGRA conversion after a median of 12.3 months in South Korea (12). As there was no control group treated with TNF inhibitors in our study, we could not directly compare the positive conversion rates of IGRA between patients treated with non-TNF inhibitors and those treated with TNF inhibitors. Nevertheless, by referring to the results of previous studies, we can indirectly infer that the frequency of positive IGRA conversion may be lower among patients treated with non-TNF inhibitors than those treated with TNF inhibitors. Considering that the risk of TB by TNF inhibitors is closely related to the local TB burden (13), our results cannot be readily extrapolated to the population of patients with IBD in other regions with different incidence rates of TB.

Out of six IGRA converters, two (Patient 3 and Patient 6) received LTBI treatment at the treating physician's discretion. As IGRA tests cannot distinguish recent from remote infections, the IGRA conversions in Patient 4 and Patient 5 were regarded as insignificant and probably associated with their previous history of TB (TB pleurisy in Patient 4 and LTBI in Patient 5). Although, Patient 1 and Patient 2 did not receive LTBI treatment, their clinical features, and serial follow-up chest radiographs did not reveal active TB development in both patients. This might be explained by test variability. As shown in Table 2, most IGRA converters had low-positive QFT results, which ranged from 0.35 to 0.99 IU/ml. Among the converters, Patient 1 did not receive any LTBI treatment, but a follow-up IGRA after 5.5 months showed reversion to negativity, and the negative result was replicated twice more after reversion (at 4.7 and 17.5 months). This suggests that positive IGRA conversion could have been due to test variability rather than true positive conversion. Another plausible explanation is the possibility of an insignificant impact of non-TNF inhibitors on LTBI reactivation. According to post-marketing data as well as vedolizumab clinical trial results and those of the IM-UNITI study of ustekinumab (7, 9, 14), it is anticipated that the risk of TB is low among IBD patients treated with vedolizumab or ustekinumab, similar to the incidence rates of TB in the respective areas of origin. This can also be supported by the Psoriasis Longitudinal Assessment and Registry (PSOLAR) data, which reported no cases of LTBI reactivation among ustekinumab-treated patients with psoriasis (15). Furthermore, previous national database study from Korea, which evaluated the risk of active TB disease among psoriasis patients treated with ustekinumab, revealed only three out of 2,803 patients (0.1%) developed active TB related to ustekinumab treatment (16). Overall, even if the cases of positive IGRA conversion were true conversions, the risk of LTBI reactivation appears to be low under non-TNF inhibitor therapy among IBD patients.

As mentioned above, there is uncertainty whether IGRA conversions represent de novo infections or reactivation of LTBI. In our study, five out of the six converters except Patient 2 who were lost to follow-up did not have any record of recent exposure to individuals with active TB. All patients underwent evaluations to exclude TB and no patient developed active TB although LTBI therapy was given to only two patients. Meanwhile, without an available gold standard test for detecting LTBI, there is also room for uncertainty resulting from the variability of IGRA results. According to previous studies on discriminating true *Mycobacterium tuberculosis* (Mtb)-specific response from test variability, 69% of borderline and 88% of low-positive IGRA results were Mtb-specific (17, 18). As shown in Table 2, except for Patient 4, all patients whose IGRA results were in the borderline zone showed a positive range of IGRA results. To overcome the inherent limitations of the test method itself, the IGRA results should be interpreted in the context of the patient's risk for TB such as epidemiologic situations. In addition, physicians should be aware of the implication of the borderline zone of IGRA value and repeat the test in cases with borderline IGRA results. The fact that we could not conduct a repeated test in Patient 4 is a limitation of our study.

In addition to the inherent limitations stemming from its retrospective design, our study was limited by its small sample size and a short follow-up duration after IGRA conversion. In particular, Patient 4 had the shortest follow-up duration because tofacitinib was initiated 2 weeks after IGRA conversion. However, even after Patient 4 switched to tofacitinib, active TB did not develop during 6.4 months of follow-up. Furthermore, we used IGRA alone to diagnose LTBI. Our domestic guidelines for LTBI diagnosis in immunocompromised patients recommend IGRA alone or IGRA combined with the tuberculin skin test (TST) (19). The TST can simply be performed *via* intradermal injection of 0.1 ml of tuberculin purified protein derivative. However, inconsistent skin reactions, subjective interpretation, and false-negative results from anergy or immunosuppressive drug use render the TST unreliable (20). Additionally, cross-reactions with bacillus Calmette-Guérin (BCG) can lead to TST positivity, and in Korea, BCG vaccination is mandatory for newborns (21). In light of these points, IGRA alone might be adequate for detecting LTBI.

Our study was the first real-life evaluation of IGRA conversion rate among IBD patients treated with non-TNF inhibitors. Further well-designed, prospective studies with larger sample sizes are warranted to identify the impact of non-TNF inhibitors on LTBI reactivation. Consequently, evidence-based, optimized strategies for TB prevention, including testing for and treatment of LTBI among IBD patients before non-TNF inhibitor therapy, could be established.

## Data Availability Statement

All data generated or analyzed during this study are included in this published article. The data underlying this article will be shared on reasonable request to the corresponding author.

## Ethics Statement

The study protocol involving human participants was reviewed and approved by the ethics committee of Asan Medical Center (IRB Number: 2021-0058). The informed consent requirements were waived by the ethics committee of Asan Medical Center.

## Author Contributions

KK and BDY: study concept and design, statistical analysis, interpretation of data, and drafting of the manuscript. KK, K-WJ, TSS, JHP, SWH, SHP, D-HY, J-SB, S-JM, S-KY, and BDY: acquisition of data. K-WJ, TSS, and BDY: critical revision of the manuscript for important intellectual content. BDY: study supervision. All authors contributed to the article and approved the submitted version.

## Conflict of Interest

BDY has received research grants from Celltrion and Pfizer Korea; consulting fees from Abbvie Korea, Celltrion, Chong Kun Dang Pharm., Daewoong Pharma., Ferring Korea, IQVIA, Janssen Korea, Kangstem Biotech, Korea United Pharm., LG Chem., Medtronic Korea, Pfizer Korea, Shire Korea, Takeda, and Takeda Korea; speaking fees from Abbvie Korea, Celltrion, Ferring Korea, IQVIA, Janssen Korea, Pfizer Korea, Shire Korea, and Takeda Korea. S-KY received a research grant from Janssen Korea. However, all of these are not related to this study. The remaining authors declare that the research was conducted in the absence of any commercial or financial relationships that could be construed as a potential conflict of interest.

## Abbreviations

IGRA: interferon-gamma release assay
IBD: inflammatory bowel disease
LTBI: latent tuberculosis infection
TNF: tumor necrosis factor
CD: Crohn's disease
UC: ulcerative colitis
IBD: inflammatory bowel disease
BCG: bacille Calmette–Guérin
TST: tuberculin skin test
Mtb: *Mycobacterium tuberculosis*.
